# Post-Operative Care of the Cancer Patient: Emphasis on Functional Recovery, Rapid Rescue, and Survivorship

**DOI:** 10.3390/curroncol30090622

**Published:** 2023-09-19

**Authors:** Victoria Morrison-Jones, Malcolm West

**Affiliations:** 1 Hepato-Biliary Surgery Unit, University Hospitals Southampton, Tremona Road, Southampton SO16 6YD, UK; victoria.morrison-jones@uhs.nhs.uk; 2 Cancer Sciences Unit, Faculty of Medicine, University of Southampton, Southampton SO16 6YD, UK; 3 Complex Cancer and Exenterative Service, University Hospitals Southampton, Tremona Road, Southampton SO16 6YD, UK; 4 NIHR Southampton Biomedical Research Centre, Perioperative and Critical Care Theme, University Hospitals Southampton, Tremona Road, Southampton SO16 6YD, UK

**Keywords:** perioperative medicine, enhanced recovery, failure to rescue, functional recovery, survivorship

## Abstract

A cancer diagnosis and its subsequent treatments are life-changing events, impacting the patient and their family. Treatment options available for cancer care are developing at pace, with more patients now able to achieve a cancer cure. This is achieved through the development of novel cancer treatments, surgery, and modern imaging, but also as a result of better understanding treatment/surgical trauma, rescue after complications, perioperative care, and innovative interventions like pre-habilitation, enhanced recovery, and enhanced post-operative care. With more patients living with and beyond cancer, the role of survivorship and quality of life after cancer treatment is gaining importance. The impact cancer treatments can have on patients vary, and the "scars" treatments leave are not always visible. To adequately support patients through their cancer journeys, we need to look past the short-term interactions they have with medical professionals and encourage them to consider their lives after cancer, which often is not a reflection of life before a cancer diagnosis.

## 1. Introduction

Cancer is reported as the leading cause of death globally, with an estimated 10 million deaths worldwide in 2020, with the world health organisation predicting this will rise to 13 million deaths globally in 2030 [1].

The aim of cancer treatment is to achieve a cure. Where this is not possible due to either metastatic disease, patient frailty, or disease burden, the focus often turns to life-extending and symptom-reducing treatments. We can divide cancer treatments into traditional, advanced, or novel categories [2]. For many years, the traditional treatment options have included surgery, cytotoxic chemotherapy, and radiotherapy. These can be either standalone treatments or used in combination [3]. Worldwide, trials are focusing on understanding the molecular mechanisms underpinning oncogenesis. There has also been some progression in advanced therapies, for example, anti-angiogenic therapy. Angiogenesis is the formation of new blood vessels from mature networks. This happens in both physiological and pathological processes within the body. Tumour angiogenesis is a key factor associated with tumour growth, progression, and metastasis; this occurs through degradation of the basement membrane, activation, proliferation, and migration of endothelial cells growth.

It is thought to be the hypoxic environment in malignant cells that induces factors such as hypoxia-inducible factor (HIF) [4]. High expression of this protein upregulates the expression of VEGF and other angiogenenic factors [5]. Anti-angiogenic therapy aims to disrupt this process, thereby inhibiting tumour growth, and it has been used in a wide range of malignancies such as colorectal [6], renal [7], lung [8], hepatocellular [9], and breast cancers. The side effects of this treatment can include high blood pressure, bleeding, and wound-healing complications. If patients are undergoing surgical intervention, this can obviously have an impact on post-operative complications.

While anti-angiogenesis is a treatment for organ malignancy, there are novel therapies being developed for haematological malignancy, for example, stem cell therapy. Stem cell therapy has a primary role in the context of haematopoietic stem cell transplantation (HSCT), where the primary goal is to replace damaged or cancerous bone marrow cells allowing for the regeneration of a functional immune system and blood cell production. Nanoparticles have also had a developing role. These structures range from 1 to 100 nanometres with a high surface-to-volume ratio. This mechanism offers several advantages in treating malignancy [2,10]. The small particles can be loaded with chemotherapy drugs; this targeted delivery enhances the efficacy while decreasing unfavourable side effects. These changing treatment modalities will change cancer treatment greatly.

Alongside understanding cancers on a molecular level, advances in surgical techniques have enabled innovation and increased the complexity of surgical operations. With this complexity comes longer operative times and increased tissue disruption, which leads to an inevitable increased physiological burden to the patient. Both prospective and retrospective studies have identified operative duration as an independent risk factor for post-operative complications [11]. These complications include surgical site infection (SSI) [12], venous thromboembolism (VTE) [13], hematoma formation, and tissue necrosis [11].

The catabolic response to major surgery is widely accepted [14], and, with more complex surgery being embarked upon, there is a growing evidence base focusing on reducing the physiological stress response in patients and improving surgical outcomes [15]. The integrated input from various medical specialities and disciplines has led to the developing field termed perioperative medicine.

## 2. Perioperative Medicine

**Figure 1:**
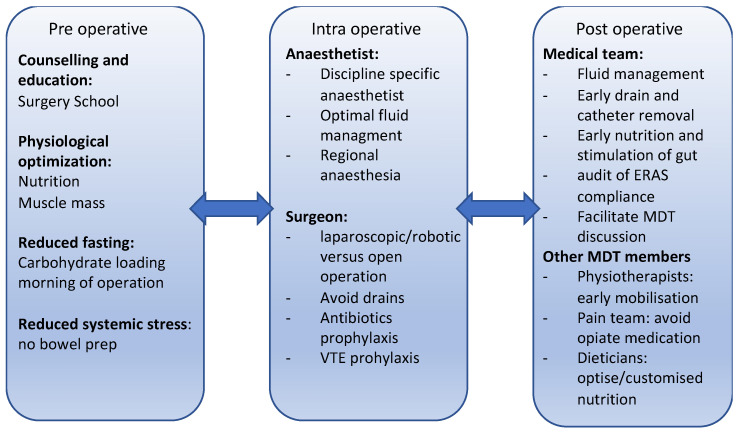
The evolving role of perioperative medicine. The evolving field looks to optimise patients pre-operatively to reduce the stress response of operations while attention to fluid management and post-operative care reduces post-operative complications and reduces post-operative stay. This involves members of the wider MDT, and there is a need to audit the data to continue improvements in this area.

Perioperative medicine is a growing, evidence-based practice where individualised, procedure-specific models of pre-and post-operative care are used to improve outcomes for patients (Figure 1). The aim is to prevent or minimise the severe detrimental effects of major cancer surgery [16,17].

There is a wealth of data published identifying factors around the perioperative period that impact post-operative outcomes for patients, many of which may be heightened in cancer patients who undergo surgical intervention. These include nutrition [18,19], functional status of the patient [20,21], the effects of adjuvant and neoadjuvant therapies [22], and the altered wound-healing commonly experienced in this group [17]. 

Although there are well-documented benefits to pre-habilitation and perioperative medicine [16,23], hospital systems are complex. For this multimodal, protocol-based practice to be implemented, a dedicated team approach requiring multiple members of the MDT is essential; thus, innovation of the health service is required [24]. Despite the current crisis of understaffing and stretched budgets within many healthcare systems, there are centres that have reported successfully implementing key components of evidence-based protocols with good results [16,24]. 

Melgar et al. published their findings after the implementation of an enhanced recovery program. They report that one-third of patients who underwent liver transplantation experienced better post-operative renal function, reduced post-operative hospital stays, and reduced incidents of readmission [25].

In 2019, the Greater Manchester Integrated Care System implemented the United Kingdom’s first multi-modal pre-habilitation (prehab) and recovery programme for cancer patients. An independent evaluation to establish the impact Prehab4Cancer (P4C) had on patient outcomes, as well as pathway and service efficiencies, demonstrated improvements for both patients and hospitals alike. A total of 1066 cancer patients who had undergone P4C were matched to a control group of non-prehab cancer patients that had undergone similar surgical interventions. This review demonstrated P4C patients had better one-year survival rates and improvements in quality of life and functional ability post-operatively when compared with non-prehab patients. In addition to patient outcomes, the hospital reported reduced post-operative stays, shorter critical care admissions, and fewer emergency readmissions. It was calculated, when based on 1000 participants, that the P4C service provision gave a threefold return on investment [26]. 

Another aspect of perioperative medicine is the implementation of enhanced recovery after surgery (ERAS^®^). The was first described as “fast-track” surgery in 1997 by Danish surgeon Professor Henrik Kehlet [27]. In this landmark paper, Kehlet proposed multimodal interventions may lead to major reductions in both morbidity and mortality in the post-operative period. He further went on to develop pre-, intra- and post-operative surgical protocols to address identified risk factors [27]. ERAS has become a critical part of surgical care. As such, in 2001, an ERAS Study Group was assembled by Professor Ken Fearon from the University of Edinburgh, UK, and Professor Olle Ljungqvist from the Karolinska Institute, Sweden [28]. Through research, collaboration, and audit, the study group identified vast differences in post-operative surgical practice across hospital units, despite some protocols already being accepted as best practice [28,29]. 

The development of the ERAS guidelines was intended to standardise models of care, reducing hospital variation and, consequently, result in a reduction in post-operative complications, reducing hospital stay and increasing patient satisfaction. 

The aims of ERAS are to

- decrease the metabolic stress and endocrine and inflammatory response to surgery.
- standardise and optimise peri-operative care across different institutes.
- decrease hospital stay, immobility and, therefore, hasten the return to normal function.
- promote pain control and decrease immobility-related complications.
- recommence enteral feeding.

The first evidence-based consensus protocol was published by Fearon et al., 2005 [29]. Since this publication, relating to patients undergoing colonic surgery, ERAS programs have been adopted as the standard of care and best practice in many surgical specialities. However, there is significant variation across to world [30,31]. With such clear post-operative protocols, one may postulate that patients who are not progressing at the expected rate may be identified by the managing team. In this, there is a shift away from “reactive” to proactive management of post-operative complications [32]. Unfortunately, large gaps still exist between what the evidence suggests should happen and what actually happens. Therefore, it is imperative that surgical teams audit their data [33] regarding compliance with ERAS pathways and determine which aspects are not being implemented [34,35]. 

Undoubtedly, surgery has become “safer”, as outcomes of surgery have improved substantially over the past decade [36]. Ghaferi published a study in 2009 where the group reviewed 84,730 patients who had undergone surgery. They found that hospitals that were reporting a low or high mortality rate had similar overall rates of post-operative complication [37]. This led to the hypothesis that the complication is not the factor causing the mortality, it is what is done about it when and if complications occur. In short, it is the failure to rescue the patient that is the principal factor in mortality rates. There have been many studies to determine the factors that contribute to this phenomenon.

## 3. Is There a Link between Failure to Rescue and Post-Operative Survival?

**Figure 2:**
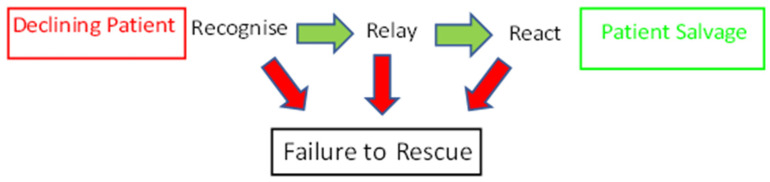
Failure to rescue. Failure to rescue (FTR) is failure or delay in recognising and responding to a hospitalised patient experiencing complications from a disease process or medical intervention. This situation leads to unnecessary mortalities. There are 3 key areas in recognising this situation and possibly reversing the situation. Early warning systems, good communication frameworks, and specialised teams have all been introduced to help reduce these mortalities.

Failure to rescue (FTR) was first described in 1992 [38]. Silber et al. reviewed a series of roughly 6000 cholecystectomies or prostatectomies to determine factors leading to variability in mortality rates across hospitals in America (Figure 2). They reported the number of post-operative deaths of patients with complications was linked to the characteristics of the hospital rather than those of the patient. The factors that have been identified as having the most significant impact are hospital surgical volume [39,40], teaching status, bed size, and intensive care unit availability [41].

Attempts have been made to reduce FTR. In 2005, the Institute for Healthcare Improvements launched its 100,000 Lives Campaign. Two key reversible events were identified during rapid clinical decline in the inpatient setting. These were a failure in adequate monitoring and a failure to respond [42]. Burke et al. have since described this as “The 3 Rs of Failure to Rescue”. Recognize, relay, and react [43]. This provides a framework of phases where interventions can occur and intercept a FTR situation. 

The implementation of escalation protocols, such as the early warning scores systems, addresses the recognise aspect. Observations that fall outside acceptable parameters trigger escalation to clinical teams and a subsequent clinical review. Communication tools such as SBAR (situation, background, assessment, recommendation) provide a framework for information handover, which addresses “relay”. The “react” is actioned by dedicated clinical teams, such as critical care outreach, who can provide experienced and timely advice in addition to medical teams. These three areas provide opportunities to intercept at reversible phases if a patient is declining.

Critical care outreach is a service that was established in Australia in the 1990s. The concept is based on Medical Emergency Teams (METs) using the well-organised principle that recognition and aggressive intervention improves outcomes from critical illness [44]. Moody and Griffiths undertook a study to determine the impact of Critical care outreach services on defined patient and service outcomes (these were length of stay, mortality, unplanned Intensive Care Unit (ICU) admissions, readmissions to ICU, in-patient cardiac arrests, adverse patient events, and severity of illness scoring). They found these services reduce ICU returns; however, there is not a “one size fits all”, and there is a significant need to review this area [44].

FTR is a factor, but the capacity to rescue is also a determinant in post-operative mortality. This capacity to rescue was first reported by the NIHR GlobalSurg collaborative. This multicentre, prospective cohort study in 82 countries showed that hospital infrastructure was essential in reducing post-operative mortality. Hospitals outside of high-income groups were less likely to have designated post-operative recovery areas where nursing-to-patient ratios are lower. Also, they were less likely to have consistently available critical care facilities and access to CT scanners around the clock. Hospitals in the low-income groups that did have access to these higher care settings had seven to ten fewer deaths per 100 major complications [3]. This confirms that, irrespective of hospital flow or bed capacity, the capacity to rescue the identified declining patient is also dependent on infrastructure and essential in reducing post-operative mortality. 

While many groups are examining what happens in FTR, there is also progress in this field through examining the care of unwell hospital patients who are successfully treated. The SUFFICE study is a mixed methods exploratory study run by Oxford University [45]. This study aims to identify common features in the care of successfully treated patients and use this to provide a framework for the escalation of care of a deteriorating patient [45]. This change in outlook will adapt our thinking from the reactive “what not to do” to the proactive “these are the steps needed”. This can only have a positive effect on post-operative mortality.

## 4. Functional Recovery after Cancer Surgery

Evaluation of functional outcome and quality of recovery (QoR) or quality of life (QoL) after surgery is a complex process, and there have been attempts to create proformas to measure this outcome. The concept is based on the resumption of several areas of normal life compared to pre-illness. These include physical [45], psychological [46], social, and economic wellbeing [47]. As a clinician, it is important to remember that different aspects of life are important to different people; this includes length of life (LoL) versus quality of life (QoL).

A study published by Meropol et al. reported that, after interviewing seven hundred and forty cancer patients, they found 55% of patients felt QoL and LoL were equally important; however, patients with advanced cancer prioritised QoL [48]. To highlight how patients can have different priorities through treatment, the National Health Service (NHS) in the United Kingdom issued guidelines on shared decision-making. This emphasises the importance of placing patients at the centre of the decision process, with their thoughts, concerns, and expectations playing a significant part in the decision-making process [49]. 

Cancer treatment is only one part of the patient’s journey. Once patients have concluded their cancer treatment, the recovery process can be critically appraised throughout its course. Function and symptoms can be assessed by patients, clinicians, and institutions by reviewing data at time points which are considered important [50]. However, the level of significance may not be the same for each group at these time points. For instance, ERAS is more clinician- and institution-focused, while an interview-based study published by Allvin et al. reported that patient focus at that time was less on total days spent in the hospital and more on returning to and continuing with everyday life [51]. It is important to address these differences and try to integrate them together. 

Efforts to define and measure the quality of recovery (QoR) and quality of life (QoL) after surgery are not new concepts [52]. The difficulty is that recovery and return to health are subjective assessments that may differ between clinician and patient and from patient to patient. Quality of recovery often relates to common adverse effects, such as pain, nausea, and vomiting, and long-term side effects of surgery. Patient-centred outcome metrics have focused on QoL [53]. A single-centre, prospective, observational cohort study of patients with high-risk upper gastrointestinal and hepatobiliary cancers demonstrated that younger patients were more willing to accept aggressive treatments that may have more adverse long-term symptoms; however, this may not be the case for older patients [54]. This may not come as a surprise, as a parent may tolerate any intervention for more time with their young children, whereas an older patient may put less value on the total number of days so long as they can stay in their own home, representing a “days in your life versus life in your days” situation.

## 5. Survivorship: Just Because It Is Gone, Does It Mean It Is over?

Earlier detection and developing treatments of many cancers have led to an increase in the number of people surviving cancer. Cancer survival in the UK has doubled over the last 40 years, with 50% of people surviving for 10 or more years; however, there remains a huge variation in cancer type [55]. Macmillan cancer support reports 3 million people living with cancer in the UK and predicts this number will rise to nearly 3.5 million by 2025, and 4 million by 2030 [56]. 

More people are living with the after-effects of cancer and its treatment, and much of the research around cancer is based on this treatment rather than its potential after-effects. This highlights the relative lack of data relating to cancer survivorship. The National Cancer Research Initiative (NCRI) has recently published Cancer Research Priorities for Living With and Beyond Cancer [57]. But research on mental health problems and challenges in long-term survivorship is still limited compared to the phases of diagnosis and acute treatment for cancer itself [58]. 

The National Cancer Institute defines survivorship as “the health and well-being of a person with cancer from diagnosis until the end of life”. This includes the physical, mental, emotional, social, and financial effects of cancer that begin at diagnosis and continue through treatment and beyond [59]. When we read about people diagnosed and successfully treated for cancer, it is easy to forget the experience is more than physical; it is not nearly as simple as “find cancer, get it out, normal life resumes”. It would be easy to presume cancer survivors only feel grateful. In some cases, this is true. However, often, the collection of emotions is as complex and unique as the cancer experience the person has lived. 

It is reported that as many as 40% of patients with cancer experience significant emotional and social distress during treatment [60], and as many as one-third of these patients may require specialist intervention [61]. However, the referral rate to these services is low, as sadness and anxiety are often perceived as “normal” responses to a cancer diagnosis. A longitudinal qualitative study reported by Cherry et al. suggested that, for some cancer survivors, participation in metacognitive therapy alleviates distress [62]. Survivorship interviews identified relief, fear, guilt, anxiety, and anger as some of the most common emotions reported. There are common themes in the anxiety reported by cancer survivors, family and finances, changes in body image [63,64], sexuality [65], and the challenges of managing long-term health. Cancer survivors may also experience challenges in returning to their pre-cancer lifestyle, including work and social activities. Fear of cancer recurrence (FCR) is the most prevalent concern reported, not only by the cancer survivor but also by spouses and immediate family members [66]. 

After active treatment, cancer patients are routinely entered into surveillance regimes. These usually take the form of consultation with the medical team, clinical examination, CT scan, routine laboratory blood tests, and, potentially, tumour markers. Unsurprisingly, FCR is reported to be heightened around the time of routine follow-up appointments. Scan-associated anxiety, or the distress before, during, or after a scan, was first dubbed “scanxiety” by a patient writing for Time Magazine in 2011 [67]. Although not an official term, scanxiety is an accepted concept; it is rational and irrational, widely spoken about by cancer patients, and reportedly unremitting. This is an example of how clinician and patient perspectives are, at times, opposing. A clinician would logically acknowledge that the further out from treatment a patient is, the better the prognosis and, therefore, anxiety at review should be lower. However, patients report being as anxious at 3 months as they are at 5 years [68]. The aim of follow-up is to assess the quality of treatment given, to give patient support, and to improve outcomes through early detection and treatment of recurrent disease. However, the evidence to demonstrate the extent follow-up has on survival remains unclear [69]. He et al. published a retrospective cohort study looking into long- versus short-interval follow-up after resected hepatocellular carcinoma. A total of 1227 patients were treated with curative intent; they found shortening the post-operative follow-up interval from every 4–6 months to every 2–4 months within the first 2 years did not prolong the overall survival of patients [70]. Therefore, due to the significant anxiety this can cause to some patients, should we be performing it, or should there be more consideration about the risk versus benefit and impact these medical interactions have on our patient’s mental health? 

Psychosocial oncology is a developing area of research; there have been many studies that have examined the potential risk factors for PTSD or associated symptoms after cancer diagnosis [71]. Powell-Chandler and colleagues reported the results collated over a two-year period of validated screening questionnaires completed by patients 12–48 months after colonic resection [72]. Multiple linear regression found that younger patients and females were more at risk of long-term psychological issues such as anxiety, depression, and PTSD [55].

Research on gender differences in response to a cancer diagnosis has gained significant attention in recent years [73]. While cancer affects both men and women, studies have highlighted distinct variations in how individuals of different genders cope with and respond to the diagnosis, treatment, and survivorship experiences [74]. The researchers found a few key areas of difference. Men are less likely to express their emotions than women, and women are more inclined to seek support from family members or friends, whereas men are more dependent on healthy spouses [74]. While gender differences are significant, they should not overshadow the importance of individualized care. Each person’s response to a cancer diagnosis is influenced by various factors beyond gender. These include age, cultural background, socioeconomic status, and personal preferences. Therefore, the circumstances of each person should be considered during their cancer pathway, and these may change through the course of their treatment.

It is also important to consider that cancer does not just affect the person with the diagnosis. It has a ripple effect on others around the person with the diagnosis. Interviews with cancer survivors indicated common areas of anxiety. Many identified that talking with family members about cancer and its treatment was challenging. Deciding whether to tell others about the diagnosis and finding an appropriate way to do so if they wanted was another concern. But, by far, the area where most patients with cancer or who have survived cancer expressed concern was worrying about how family members were coping emotionally with everything that had happened. They often felt it was harder on the family than on themselves [75].

There are now more support systems in place for not just the patient. Children with parents that have cancer can now access specialised support. Spouses can have dedicated space where they can express their feelings and support, where families can be helped with how best to communicate and reduce many of the common anxieties each party feels.

## 6. Discussion

A cancer diagnosis is a life-altering event that disrupts every aspect of a person’s life and the lives of those around them. It represents a new state of being, where nothing can be taken for granted. Significant progress has been made in improving cancer survival. The survival data for some cancers are more promising than a decade ago, but variation in regard to tumour type remains. The progression in treatment options and, therefore, in the number of cancer survivors is a testament to the cancer research carried out across the globe.

As the number of patients living past cancer is increasing, improving patient quality of care during and after cancer treatment has never been so important. But there is little consensus on how best to achieve this. There needs to be a growing focus on survivorship care that aims to provide support beyond the initial treatment phase.

The scars that cancer treatment can leave are not always visible; therefore, addressing mental health and understanding the long-term impacts of cancer and its treatments is vital. We have to change our mindset. Cancer treatment goes beyond mere survival; it is also about ensuring people live well and are able to pursue a fulfilling and meaningful life after their cancer diagnosis.
